# *Rickettsia rickettsii* Transmission by a Lone Star Tick, North Carolina

**DOI:** 10.3201/eid1705.101530

**Published:** 2011-05

**Authors:** Edward B. Breitschwerdt, Barbara C. Hegarty, Ricardo G. Maggi, Paul M. Lantos, Denise M. Aslett, Julie M. Bradley

**Affiliations:** Author affiliations: North Carolina State University, Raleigh, North Carolina, USA (E.B. Breitschwerdt, B.C. Hegarty, R.G. Maggi, D.M. Aslett, J.M. Bradley);; Duke University Medical Center, Durham, North Carolina, USA (P.M. Lantos)

**Keywords:** ticks, disease, rickettsia, rash, thrombocytopenia, bacteria, lone star tick, North Carolina, vector-borne infections, dispatch

## Abstract

Only indirect or circumstantial evidence has been published to support transmission of *Rickettsia rickettsii* by *Amblyomma americanum* (lone star) ticks in North America. This study provides molecular evidence that *A*. *americanum* ticks can function, although most likely infrequently, as vectors of Rocky Mountain spotted fever for humans.

Historically, transmission of *Rickettsia rickettsii* has been attributed to *Dermacentor variabilis* ticks in the eastern United States and to *D. andersoni* ticks in the western United States (*1*). Recently, researchers at the Centers for Disease Control and Prevention (CDC; Atlanta, GA, USA) documented transmission of *R. rickettsii* to persons residing in Arizona by a novel tick vector for North America, *Rhipicephalus sanguineus* (commonly referred to as the brown dog tick or kennel tick) (*2*,*3*). As reviewed by Goddard and Varela-Stokes in 2009, only scant evidence has been published to support transmission of *R*. *rickettsii* by *Amblyomma americanum* (lone star) ticks in North America (*4*), although Parker et al. in 1933 reported experimental transmission of rickettsial infection by lone star ticks (*1*), and other *Amblyomma* species, such as *A. imitator* and *A. cajennense*, are likely vectors for Rocky Mountain spotted fever (RMSF) in Central and South America (*5*).

## The Study

A 61-year-old man visited his physician in May 2010 and reported a history of fevers, myalgias, and headache. He had been free of symptoms until 3 days earlier, when he experienced nausea and dizziness. The following morning, he had chills and, later the same day, a fever of 38.4°C. Mild headache and myalgias accompanied his fever. His symptoms became progressively more severe during the next 2 days, and fever reached 39.4°C.

The patient resided on a farm in central North Carolina and had occasional exposure to ticks. Seven days before the onset of illness, he had removed an embedded tick from his right axilla. He estimated that the tick had been attached for 2–3 days. Clinical examination showed an eschar-like lesion at the site of the tick bite that appeared erythematous with a necrotic center (Figure). The patient’s neutrophil count (2,553 cells/µL) was within low reference limits, accompanied by 5% band neutrophils, and mild thrombocytopenia (148,000 platelets/µL).

**Figure:**
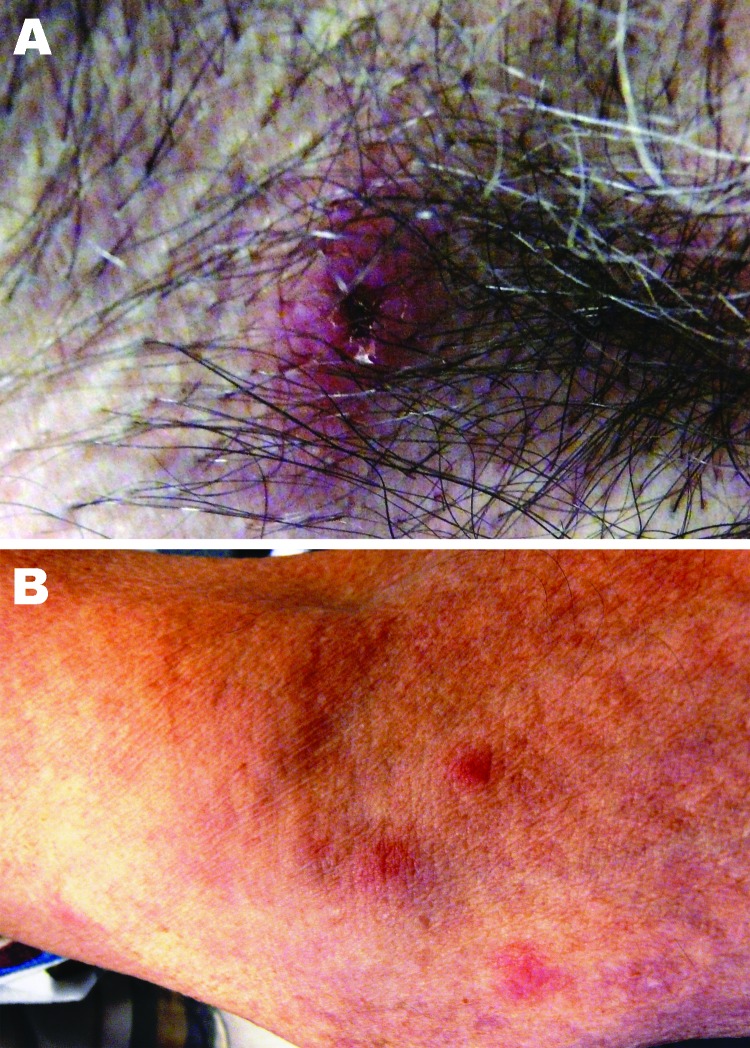
Images of lesion in the patient caused by bite from lone star tick. A) Erythematous circular lesion in right armpit at site of tick bite with induration and a necrotic center. B) Maculopapular rash involving the inferior portion of the arm. Source: Julie M. Bradley.

A tick-borne rickettsiosis or ehrlichiosis was suspected, and on the 4th day of illness, the patient was prescribed doxycycline, 100 mg 2×/d for 7 days. A maculopapular rash appeared the next morning, which predominantly involved the extremities but not the palms or soles. Fever resolved within 36 hours after beginning therapy, and the rash began to fade. Within 1 week after receiving doxycycline, the patient’s symptoms and hematologic abnormalities had resolved (neutrophil count 4,378 cells/µL, no band neutrophils, and platelet count of 320,000/µL).

The patient provided the tick, which he had preserved in alcohol. The tick was identified by using Herms’s taxonomic key to *Ixodes* spp. ticks as a male lone star tick (*A. americanum*). Amplification of the mitochondrial 16S rRNA gene confirmed the tick species as *A. americanum* (417/418 bp, 99.8% homology with GenBank sequence L34313) (*6*).

Acute-phase serum was collected and stored until the convalescent-phase sample was obtained. Subsequently, seroconversion to *R. rickettsii* antigens was documented (reciprocal acute-phase titer 64, convalescent-phase titer 512 after 4 weeks). The patient did not seroconvert to *Ehrlichia* sp. antigens.

*Rickettsia* and *Ehrlichia* spp. PCRs were performed immediately. A previously described *Rickettsia* sp. PCR (primers 107F and 299R) was used to target a 209–212-bp fragment of a variable region of the outer membrane protein (*omp*) *A* gene, with species identification determined by DNA sequencing (*7*). Amplicons were obtained from the patient’s blood and from the tick. After direct sequencing and comparative alignment with GenBank sequences, identical *R. rickettsii ompA* sequences were obtained from the patient’s blood and the tick (Table). PCR using 17-kDa primers (17k-5s and 17k-3) were used to further support infection with *R. rickettsii* (*8*). An amplicon with 100% homology (502/502 bp) with *R. rickettsii* 17-kDa surface antigen precursor (*omp*) gene, GenBank sequence AY281069, was obtained from the tick, and a 17-kDa amplicon was obtained from the patient’s blood, but the DNA concentration was too low for sequence analysis. When the *ompA* and 17-kDa sequences from the tick and the *ompA* sequences from the patient’s blood were compared with those from *R. amblyommii*, *R. parkeri*, and *R. rickettsii*, sequences matched *R. rickettsii* (Table). A PCR result for *Ehrlichia* spp. DNA was negative for samples taken from the tick and the patient.

**Table:** Sequence similarities for the *ompA* and *17-kDa* genes from *Rickettsia* spp. amplified and sequenced from the patient’s blood and from the *Amblyomma americanum* tick, North Carolina, 2010*

Characteristic	*ompA*				*17-kDa*				
	*R. rickettsii*	*R. parkeri*	*R. amblyommii*		*R. rickettsii*	*R. parkeri*	*R. parkeri*	*R. amblyommii*	*R. amblyommii*
GenBank no.	DQ002504	U43802	AY062007		AY281069	EF102237	U17008	AY375162	U11013
Tick sequence similarities, bp	180/180	171/180	159/180		502/502	496/497	486/489	480/490	479/489
Patient sequence similarities, bp	176/176	159/180	NA		NA	NA	NA	NA	NA

*ompA, outer membrane protein A gene; NA, amplicon not obtained from patient’s blood sample for sequence comparison.

According to CDC criteria, RMSF developed in this patient after he was bitten by an *A. americanum* tick. Evidence to support a diagnosis of RMSF included 1) amplification of *R. rickettsii* DNA from the patient’s blood sample at the onset of illness; 2) development of a prototypical rickettsial illness, including fever, rash, and thrombocytopenia 8 days after the tick was removed; 3) amplification of *R. rickettsii* from the alcohol-stored tick removed by the patient; 4) documentation of seroconversion to *R. rickettsii* antigens; and 5) a rapid and appropriate clinical response after treatment with doxycycline. The patient was not aware of any other recently attached ticks. Therefore, infection by an *A. americanum* tick caused by preexisting rickettsemia in the patient seems unlikely.

## Conclusions

On the basis of historical (*4*) and recent PCR data, transmission of *R. rickettsii* by *A. americanum* ticks is most likely an infrequent event in North America (*9*). However, in Central and South America, *A. cajennense* and *A. imitator* are suspected vectors of *R. rickettsii* (*5*). For clinical and surveillance purposes, it is essential to recognize that at least 3 tick genera, *Dermacentor*, *Rhipicephalus*, and *Amblyomma*, may be capable of transmitting RMSF in the eastern and south-central United States.

During the past decade, CDC has reported a progressive rise in cases of RMSF, particularly in the eastern United States (www.cdc.gov/ticks/diseases/rocky_mountain_spotted_fever/statistics.html). During the same period, there has been a concurrent expansion in the geographic range of *A. americanum* ticks in conjunction with reports suggesting an increased frequency of attachment of this tick species to animals and humans (*10**,**11*). Although *R. ambylommii* is frequently amplified from *A. americanum* ticks, no current evidence supports a pathogenic role for this *Rickettsia* sp. in animals or humans (*11*). In contrast, *A. americanum* ticks can transmit *R. parkeri*, which is a recently documented human pathogen, most often transmitted by the Gulf Coast tick (*A. maculatum*) (*12**,**13*). Eschars have occurred frequently in the limited number of *R. parkeri* cases published, whereas eschars, such as the one seen in this case, are uncommonly described in association with RMSF (*14*). Using the 180-bp *ompA* gene variable region we sequenced in this study, we differentiated *R. rickettsii* from *R. parkeri* and *R. ambylommii* by 9 and 21 bp, respectively. Substantial efforts to amplify *R. parkeri* by using multiple primer sets (data not shown) from the tick and the patient’s blood were not successful.

Taken in the context of other recent studies, this report supports the hypothesis that lone star ticks can transmit *R. ambylommii*, *R. parkeri*, and *R. rickettsii* infections to persons in the United States (*12**,**13**,**15*). An increasing diversity of competent tick vectors in conjunction with recent identification of novel *Rickettsia* spp. may be contributing to the increase in seroepidemiologic surveillance trends reported for RMSF. Because extensive serologic cross-reactivity exists among rickettsial species, defining the infecting species requires organism isolation in a biosafety level III laboratory or PCR amplification and DNA sequencing, as was used in this study.
